# Blockchain integration in healthcare: a comprehensive investigation of use cases, performance issues, and mitigation strategies

**DOI:** 10.3389/fdgth.2024.1359858

**Published:** 2024-04-26

**Authors:** Meenavolu S. B. Kasyapa, C. Vanmathi

**Affiliations:** School of Computer Science Engineering and Information Systems, Vellore Institute of Technology, Vellore, India

**Keywords:** blockchain, healthcare, use cases, performance issues, off-chain solutions, on-chain solutions

## Abstract

Healthcare is a critical area where blockchain technology (BT) is being heralded as a potential game-changer for facilitating secure and efficient data sharing. The purpose of this review is to examine BT applications, performance challenges, and solutions in healthcare. To begin, This review paper explores popular blockchain networks for data exchange, encompassing both public and permissioned platforms, such as Ethereum and Hyperledger Fabric. This paper analyzes the potential applications of BT’s decentralized, immutable, and smart contract capabilities in healthcare settings, including secure and interoperable health data exchange, patient consent management, drug supply chain oversight, and clinical trial management. The healthcare industry might greatly benefit from the increased privacy, transparency, and accessibility that these technologies provide. Despite BT’s promising medical uses, the technology is not without its drawbacks. High energy consumption, throughput, and scalability are all concerns. We wrapped up by discussing the solutions that have been implemented, including consensus processes, scalability measures like sharding, and off-chain transactions that are designed to mitigate the drawbacks.

## 1 Introduction

The healthcare industry is constantly on the lookout for ways to improve the safety and efficacy of its data management methods in the modern digital era. With increasing digitization and a vast population, data management and safeguarding sensitive patient data have become paramount (1). Traditional centralized database systems will no longer be able to meet the complex requirements of healthcare organizations, like data sharing with security and privacy (2). So, healthcare organizations aim to improve these by adopting innovative solutions (3). In this pursuit, centralized SQL databases were replaced with cutting-edge technologies such as cloud computing and automation. Cloud-based (4) and big-data-based (5) systems offer scalable and flexible storage solutions, enabling healthcare organizations to efficiently manage and access vast amounts of patient data. Automation could streamline monotonous tasks like data input and report preparation, freeing up doctors to focus on patient care.

Cloud computing and automation can enhance healthcare data storage, retrieval, and analysis productivity, but security and privacy must be prioritized. To prevent patient data breaches, strong security procedures have been adopted. Encryption, access limitations, and multi-factor authentication are used. Furthermore, powerful threat detection tools and regular security audits find security weaknesses. Healthcare data management benefits from machine learning (ML) and artificial intelligence (AI) integration. Algorithms powered by ML (6) and AI (7) can find patterns, predict disease outcomes, and aid in medical decision-making by analyzing vast patient data sets. Despite these advanced methods, healthcare data transparency, ownership, and privacy remain the biggest concerns (8). Blockchain technology provides a decentralized, secure, and transparent platform for storing, sharing, and managing health data (9).

Blockchain is simple but revolutionary. Bitcoin (10) and Ethereum (11) can transform healthcare. Instead of storing each record on a vulnerable server, it is spread across several PCs worldwide. This distributed ledger technology eliminates single points of failure and increases privacy controls over sensitive medical data like electronic health records (EHRs). Thus, BT can improve healthcare by boosting security, transparency, and efficiency. Despite its numerous advantages, integrating this technology into current infrastructure is difficult.

The purpose of the review paper is to investigate the impact of BT on the healthcare sector and its potential uses. Later, it explores the potential impact of BT on the health industry. Difficulties and challenges with blockchain healthcare integration, and finally, we will conclude by discussing the proposed mechanisms to overcome these difficulties.

## 2 Background work

### 2.1 Healthcare data security

Healthcare data like EHRs and personal health information (PHI) require significant security measures. Protecting sensitive patient information, preventing its unwanted use, and preventing theft are all aspects of healthcare data security. Countries throughout the world have established several laws and regulations in an effort to ensure the security of this information, like the India Digital Personal Data Protection Bill and the United States Health Information Technology for Economic and Clinical Health (HITECH). Table 1 provides a summary of these regulations and laws.

**Table 1:** Healthcare data management regulations and laws.

Country	Regulation or law	Key provisions
India (12)	The digital personal data protection bill	Digital data privacy guidelines were initially introduced in 2000 and revised several times. The most recent revision was in August 2023.
United States (13)	Health insurance portability and accountability act (HIPAA)	HIPAA was introduced in 1996 to create standards for EHR and PHI protection. In 2009, the HITECH Act was introduced as an extension to HIPAA to strengthen privacy and security provisions.
European Union (14)	General data protection regulation (GDPR)	Regulations for the protection of personal health information, applicable across all EU member states, came into force in 2016.
Canada (15)	Protection and electronic documents act (PIPEDA)	Guidelines for the privacy, utilisation, and availability of individual information came into existence in the year 2000.
Australia (16)	My health records act	Introduced in 2012 to ensure patient ownership of their health records and enable the my health record system to store, utilize, and disclose them.

Despite the extensive framework that different countries’ laws and regulations have provided for protecting personal information, there is still a problem. Due to the sensitive nature of healthcare information, there is a high demand for it on the dark web, and attackers are willing to pay a high price for it. Names, ages, addresses, Aadhar numbers, bank account numbers, and other private information are all examples of what falls under this category. Because of this, the primary objective of the healthcare industry is to create a plan that is both effective and efficient for ensuring data is kept as secure as possible. Access control and data encryption are offered for EHR data security throughout the first stages of healthcare application development. Rivest-Shamir-Adleman (SHA), Data Encryption Standard (DES), and elliptic curve cryptography (ECC) are popular data encryption algorithms (17). Creating role-based access control policies is a common approach to authenticating for access control (18). in later years to improve patient care. Medical IoT devices have been integrated (19), and to solve these complex structure and performance issues, a cloud environment is used for storing the data (20). In this new approach, data mobility and data security are key issues (21). This problem leads to the invention of new security approaches like the double-layer security approach for data (22) and hiding schemes for medical images (23). Even though many methods are available for medical data security, EHR privacy and ownership are still the main issues, and data access is not controlled by patients. It will be solved with the inclusion of blockchain (24).

### 2.2 Principles of blockchain

Blockchain is a subset of decentralized distributed ledger technology (DDLT), in which each participant acts as a node in the network. Rather than traditional centralized structures, each node in this network establishes an independent connection to one another, much like a P2P (peer-to-peer) network. In this P2P network, all interactions take the form of transactions, which are then grouped together to form a node. This block is shared across the network with every user, which increases the safety of the system because any alteration would have to be made to every user’s copy of the ledger, which makes tampering impossible. So once a transaction has been recorded in the blockchain, its immutability guarantees that it cannot be changed. Data encryption and decryption via algorithms like SHA256 and ECC strengthen its security and privacy. The public can view all of the transactions that have been recorded on a blockchain, which builds trust and reliability. These characteristics have attracted enthusiasts from a wide range of industries, like digital currencies, data sharing, and supply chain management.

Since each node holds an identical copy of the data chain, blockchain networks provide data redundancy and decentralization. Maintaining consistency across all nodes is tough because each node contains the same data chain. An agreement on which block data to include in the chain at each block addition ensures consistency. This is possible with consensus protocols. Some of the popular consensus algorithms are POW (proof of work), POS (proof of stake), the hybrid model (POW+POS), PBFT (practical Byzantine fault tolerance), and PoET (proof of elapsed time) (25). In public networks like Bitcoin and Ethereum, every block contains the following mandatory components: Version (the current version of the block structure), Nonce (number used once randomly selected at creation), Previous block header hash, Merkle root hash (cryptographic hash of all the transactions recorded in the block). Timestamp (block-created time).

The application, middle, consensus, network, and data layers are usually observed in blockchain networks. The application layer contains user interface applications and smart contracts (26). This layer is responsible for providing flexibility for users to do transactions. These transactions moved to the middle layer. Based on the platform, transactions are processed in this layer. After being processed, they advance on to the consensus layer. This consensus layer validates and verifies transactions and also helps to maintain consistency in the network. So, in the consensus layer, transactions are verified and bundled in the Merkle tree structure (27) to create a new block. After validation, this new block is sent to the network layer for distribution to every node in the entire network. This block will be validated at every node. After successful validation, the ledger will be updated and sent to the data layer. The data layer is responsible for storing data on every node in the network alongside the rest of the blockchain, guaranteeing data redundancy and decentralization. and finally storing the data at every node. Consensus algorithms are essential to blockchain network credibility. These algorithms work across layers. It decides whether transactions are valid, in what order to include blocks in the consensus layer, and who has authorization to produce the block. In the network and data layers, it plays a very little role.

**Figure 1:**
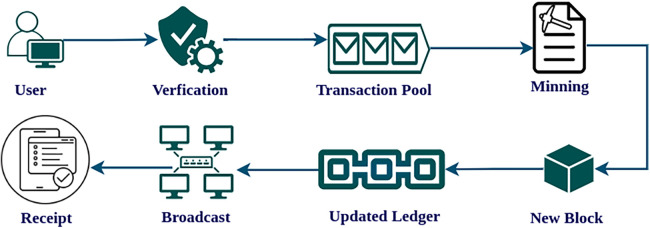
Blockchain transaction processing.

The general framework for transaction processing in a blockchain network is shown in Figure 1. It begins with transaction initiation, where a user creates a transaction by specifying the amount to be transferred and the receiver address. Next, the network validates the transaction by verifying the sender’s identity and fund availability. Later, verified transactions are sent to the transaction pool. Then miners or validators engage in mining or consensus, competing to establish a new block of transactions. After block creation, block confirmation follows, where nodes confirm the block’s accuracy and conformance to regulations. Upon confirmation, the blockchain’s ledger is updated. This updated ledger is broadcast and stored at each node in the network. Finally, transaction completion is done. Upon completing the transaction, users obtain transaction receipts as verification of their transactions, while miners collect block rewards (depending on the blockchain network and consensus mechanism), and the recipient obtains access to the transferred funds. This process supports the functioning of blockchain networks, maintaining the integrity and transparency of transactions.

### 2.3 Blockchain for data sharing

At first, blockchain was implemented in Defi (decentralized finance) apps to ensure the safety of financial activities. Later, as a result of its advantages, data transfer became significantly in demand. Bitcoin (10), the first blockchain network, was only used for crypto exchange, so new platforms have emerged for data sharing. Especially after the introduction of Ethereum, usage of blockchain has increased beyond crypto currency.

Ethereum (11, 28) is one of the earliest and most well-known blockchain platforms, having first appeared in 2013. Ether (ETH) is the cryptocurrency used on the Ethereum network. It’s the first model for maintaining data exchanges, and it’s a decentralized network that anyone can join. The ability to view and modify your own data-sharing preferences is a major benefit. It also allows for the development of smart contracts, which can be used to automate getting out of an agreement. Smart contracts make asset management simpler than the Bitcoin blockchain’s scripting language ever could. In addition to allowing existing business logic to be extended, these smart contracts also allow for integration with a wide variety of programming languages and tools. Transactions in Ethereum are signed data packages to be sent from an externally owned account Containing recipient, sender signature, amount (ETH), the data to send, and the maximum fee in wei for transaction processing. Including data in the transaction is optional. Wei is the smallest value in Ethereum. Transaction validators are compensated for tasks completed.

Hyperledger Fabric (29, 30) launched in 2016 as an open-source permissioned blockchain. It is one of the products of the Hyperledger family. It is the combined work of IBM, the Linux Foundation, Intel, Digital Asset, and Accenture. Fabric is the first product of this consortium. This platform is used to create applications and solutions using a modular architecture. Since it is a private blockchain, participants need to be authorized to join the network. Every member of the network can authorize nodes to participate with the help of the fabric certificate authority. Usually, a predefined group of people confirm transactions, but they can alternatively be confirmed by mutual consent between the sender and the recipient. You can create your own consensus. On this platform, smart contracts can be called chain codes. This chaincode is responsible for the consensus mechanisms for their Dapp. It is not providing any networks. The developers need to deploy on their own space or servers because it has no native cryptocurrency, no mining fee, and no consensus. As a result of their network, only members of the network can see the operations; other application users cannot access the data, unlike Ethereum. Due to these features, transaction speed is faster, so performance and scalability are very high compared to Ethereum.

MultiChain (31, 32) is also one of the private blockchain platforms. It provides a simple environment to configure and deploy packages. It supports all types of servers and allows integration with existing systems by generating a JSON-RPC API. This platform’s special feature is that it allows only selected participants to monitor blockchain activity. On this platform, developers can create data streams (33) that allow only selected types of transactions in the network. Administrative privileges on the network are automatically allocated to Genesis miner. This administrator selects miners who produce blocks based on the mining diversity, which has a value between 0 and 1. A diversity number of 0 allows any miner to create a block at any time, but a value of 1 requires block production using the round-robin mechanism. According to the developer 0.3 is the default value but 0.5 or greater is the strong mining diversity. Multichain allows participants to customize the blockchain’s parameters (34) in a configuration file. Due to this feature, scalability problems can be solved. It allows developers to create their own cryptocurrency and also allows other cryptocurrencies to be used as tokens.

Corda (35) is an innovative private blockchain platform that allows blockchain interoperability, enabling different networks to communicate to exchange data. It has the potential to be immensely useful in the fields of digital assets, healthcare, insurance, capital markets, and government. It is unquestionably perfect for enterprises as an open-source blockchain platform with considerable flexibility. In terms of consensus and currency, it is identical to the hyperledger fabric. Corda smart contracts are not as fully functioning as other platforms, but they will carry out the required functions and are written using Java. By default, the Corda platform includes packages that support Oracle services. The Hyperledger consortium released support for Hyperledger Indy’s interface with Corda.

IOTA’s Tangle framework (36) is the first decentralized, cost-free distributed ledger designed specifically for IoT device communication. It simplifies and speeds up IoT device data sharing and financial transactions. Unlike other blockchain protocols, IOTA does not charge transaction fees (37). This makes it a good option for storing large files, like images. It can execute many transactions per second. Making it ideal option for achieving high-throughput and scalability for applications use image and videos. In addition number of open-source tools and packages are available to support easy image processing. For instance, the IOTA Foundation has released a JavaScript framework called the IOTA Streams Framework that facilitates data encryption and storage on the IOTA Tangle. Compared to Ethereum, IOTA Tangle requires longer to build an ecosystem of apps and services.

BigchainDB (38) combines blockchain technology with a distributed database architecture. It seeks to provide scalability, high throughput, low latency, and querying and indexing capability, which Bitcoin and Ethereum struggle with. It increases scalability by making trade-offs compared to typical blockchains. This platform does not use full replication, so each node does not need to store the entire blockchain. This decreases BigchainDB node storage and bandwidth. Its consensus algorithm differs from that of standard blockchains. BigchainDB’s Byzantine fault-tolerant (BFT) consensus process allows it to function even with one-third of the nodes offline. This strengthens BigchainDB against attacks and failures. Though still in development, it might be a great tool for many applications.

Kadena (39), Solana (40), EOS (41), Eris (42), and LUCE (43) are a few examples of other potentially useful platforms for the exchange of data.

## 3 Blockchain in healthcare

The healthcare system has changed in recent years to support a patient-centered approach. To achieve this, the health industry is exploring the potential of BT for managing health records. Because BT is a decentralized, secure, and transparent system, it can help address these new problems. Several studies have shown that BT has the potential to improve healthcare data management. Here, we summarize the literature about blockchain health record management and discuss its potential applications and drawbacks.

For example, Azaria et al. (44) presented the BT framework Medrec for maintaining EHRs. It provides a secure and decentralized solution to storing and exchanging patient data, giving patients authority over their data while also allowing healthcare professionals to access and interchange it safely and promptly. Similarly, Kshetri (45) highlighted that BT has the ability to improve the security and privacy of healthcare data while also lowering medical errors and fraud in the supply chain. Omar et al. (46) conducted a study that emphasized the use of BT in clinical trials for data authenticity, transparency, and traceability.

### 3.1 Use cases of blockchain technology in healthcare

**Figure 2:**
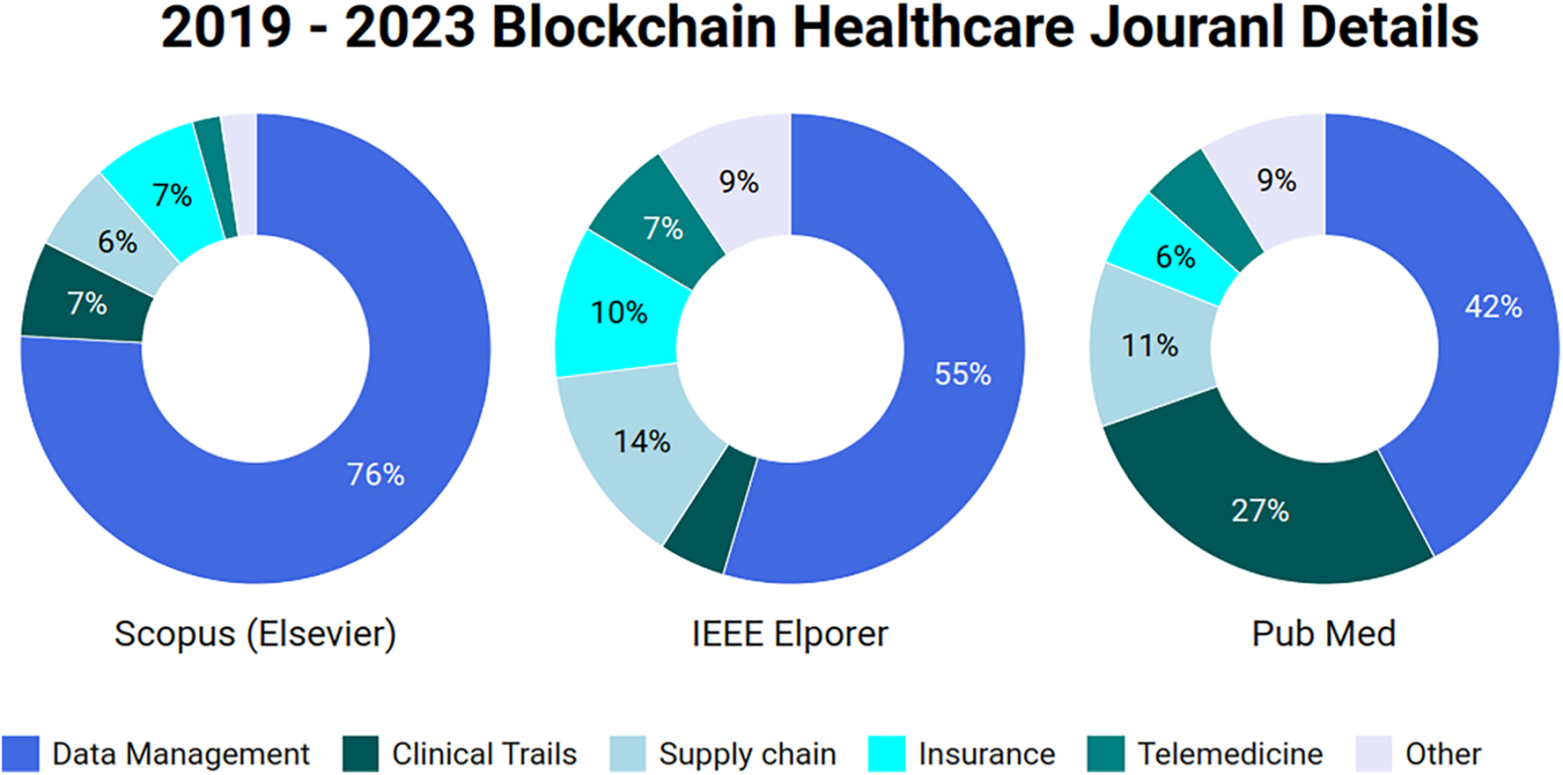
Published papers data on healthcare blockchain in 3 different databases.

In our comprehensive analysis of blockchain applications in healthcare across several databases, we uncovered a significant quantity of research on this rapidly expanding topic. We identified 3,800 records connected to blockchain healthcare in Scopus (Elsevier), 1,383 in IEEE Access, and 537 publications in PubMed as of September 2023. This comprehensive collection of literature highlights the growing interest in and importance of blockchain technology in tackling healthcare concerns. Figure 2 shows that researchers have prioritized crucial areas, including healthcare data management, clinical trials, supply chain management, and insurance claims applications. These domains have emerged as key use cases for blockchain in the healthcare industry.

To identify relevant publications within each domain, we used category-specific keywords. For instance, in the context of BT in healthcare we utilized phrases like “blockchain” and “healthcare.” In the context of EHR management, chosen keywords were “data management,” “blockchain,” and “healthcare.” Similarly, for clinical research, we employed the terms “blockchain” and “clinical research,” while “healthcare” and “supply chain” were used for BT for supply chain. and finally for healthcare insurance, relevant terms encompassed “blockchain,” “healthcare,” and “insurance.” To preserve the dataset’s integrity, our study retained duplicate contributions and presented it exactly as retrieved from journal databases. Although the presence of duplicates in the dataset may have an influence on certain studies, we choose to keep them since we value transparency. This decision illustrates our dedication to openness and transparency in our research process.After displaying the dataset in a figure, we used keywords and abstracts to select the 400 notable papers. We carried out a comprehensive screening process to ensure the quality and rigor of our investigation. Every study was meticulously examined to ensure its pertinence and quality, aiming to include only relevant material in our analysis.

#### 3.1.1 EHR management

**Figure 3:**
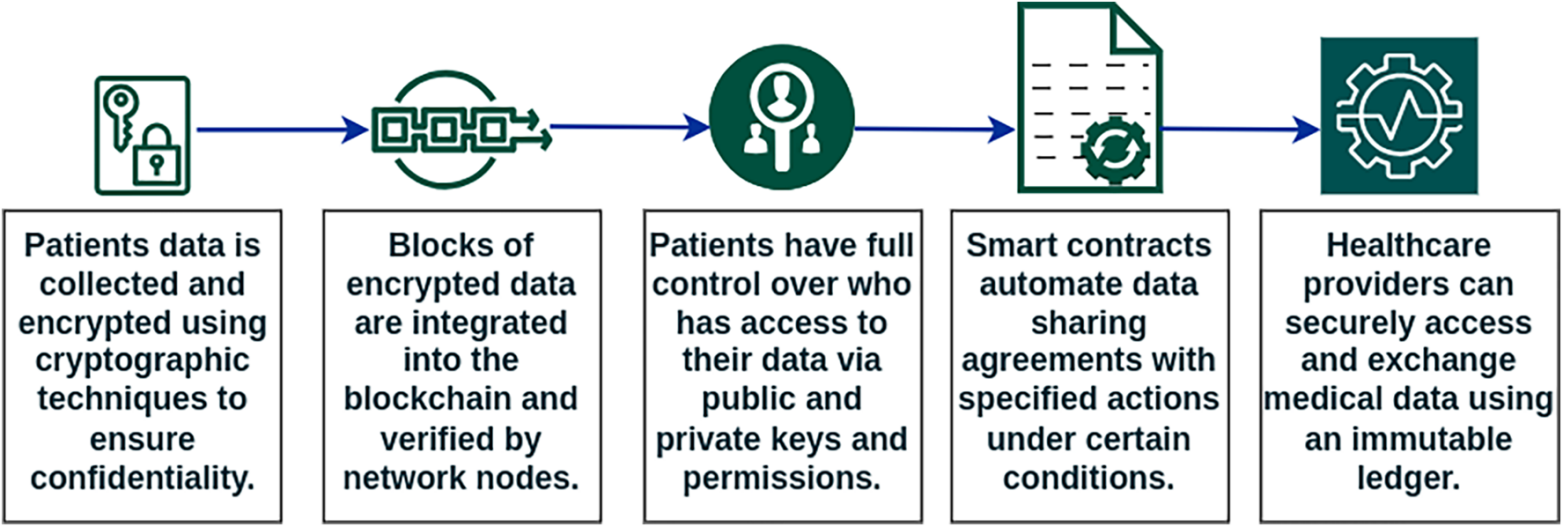
Health record management with blockchain.

EHR data management is critical in medical care, and BT provides various benefits to the healthcare industry. It simplifies operations by allowing patients to control and securely exchange medical records with their preferred medical professionals. Encryption technology protects security, and limiting access to authorized users provides privacy and prevents hacker attempts. This lowers unnecessary paperwork for patients, improves healthcare delivery, and reduces data loss concerns. The process of managing healthcare data with BT has been illustrated in Figure 3. Key ways in which BT can be used to improve EHR management include secure data storage, patient control, interoperability, and integrity.

**Secure data storage:** Data is hard to hack due to BT’s decentralized architecture. Data is kept on several computers throughout a network, not in one place. Another key aspect of BT is immutability. It ensures that ledger data cannot be manipulated without discovery. This feature protects against data modification and fraud.

**Patient control:** BT gives patients full ownership over their medical records. Patients can choose who sees and uses their data. A hash value is formed when data is saved in the blockchain. This hash stores data location and approved users who can read or alter it. This data will instruct blockchain apps. So when doctors, nurses, patients, or other permitted users or devices seek patient file information, the program validates their credentials before giving or denying access. Data access control is transparent and does not divulge sensitive information to unauthorized people with this strategy. These programmers record all data interactions for auditability. Every blockchain platform provides programs for this. Ethereum-like platforms employ smart contracts to control data access.

**Interoperability:** BT incorporation enables healthcare system interoperability. This can improve care coordination and prevent medical errors. The decentralized technology eliminates intermediaries in patient data sharing between institutions and medical practitioners. This simplified interchange improves care coordination and patient outcomes.

**Integrity:** Since each transaction or event has a unique hash, BT guarantees EHR data integrity. Future users can verify content integrity by recording the hash. Updated documents generate different hashes that don’t match the original hash code.

Sharma et al. (47) developed a framework with Hyperledger Fabric for fundamental EHR management features. While this framework does make it easy to enter EHR data, it lacks in terms of who has access to that data. Similarly, the QI XIA et al. (48) healthcare platform is limited to established cloud service data providers. Smart contracts on this Ethereum-based platform use private keys to ensure the authenticity of data transfers in the healthcare industry. Patients do not have access to their information in this study. There are serious concerns about patient privacy with these solutions. Giving complete access control to patients is one of the suggested solutions for enhancing patient data privacy. Omar et al. (49) described a blockchain-based system for storing and sharing private medical records. Data is encrypted in a federated blockchain, and access to the secret key requires permission from the data’s owner. Dubovitskaya et al. (50) created a framework especially for cancer patients with Hyperledger Fabric and EHR data stored and managed in a HIPAA-compliant cloud with the help of Amazon Web Services (AWS). Haritha et al. (51) presented a framework for securely accessing the levels of health records in an organization using lattice-based access control in a smart contract blockchain platform.

Several other researchers also presented healthcare environments and frameworks. Vora et al. (52), Jabbar et al. (53), and Chamola et al. (54) created Ethereum-based privacy models. Liang et al. (55) and Munoz et al. (56) created the Hyperledger privacy solutions. Xu et al. (57) created a custom blockchain solution. Abdullah et al. (58) proposed an IOTA-tangle blockchain-based solution. An overview of EHR management with BT is presented in Table 2. These frameworks enhance security and privacy in healthcare environments. The majority of frameworks focus on essential healthcare data transfers, allowing for simplified and secure data sharing among authorized parties. They also have the ability to address difficulties in ML and AI-based systems, notably those involving data security and privacy. Chen et al. (59) proposed a blockchain framework called Learning Chain to overcome data privacy and security issues in traditional centralized ML applications. It facilitated a decentralized and secure environment for training ML models while protecting data privacy. Although ML and AI help healthcare BT apps, some researchers have highlighted ML and AI integration with blockchain for healthcare. Cheng et al. (60) discussed ML and BT integration advantages in cancer care. Nguyen et al. (61) presented a scenario of AI and BT for fighting the COVID-19 epidemic. Chamola et al. (54) proposed an AI-based medical history summary generation with IPFS and Ethereum. It allows less time for studying old medical data. Kalita et al. (62) proposed a ML-based BT framework for managing maternal health information exchange. The Random Forest algorithm is used for data analysis to reduce complications of pregnancy.

**Table 2:** Comparison of EHR management applications and frameworks with blockchain.

Paper	Implementation	Benefits	Issues	Further research
(47)	Hyperledger fabric private network	Security, privacy	The patients cannot manage their data	Implementation of smart contracts for advanced EHR management features like billing are required
(49)	Framework with its own private blockchain	Security, privacy	No practical implementation	Practical aspects need to be examined
(52)	Ethereum private network	Security, ownership	Interoperability, performance	Exploring more on privacy and data models
(53)	Permissioned prototype with ethereum testnet	Interoperability, security, privacy	Restricted usage	Test on the main network
(55)	Hyperledger fabric private network	Security, privacy, latency	Participants are limited	Large-scale application scenario
(63)	Ethereum employs IPFS for image storage and a trustworthy oracle for automatic key creation	Security, cost efficient	Interoperability, inconvenient key management	Need to create a feasible global solution
(64)	Ethereum network, clouds for image storage with ECC & EdDSA for encryption	Security, cost efficient	Performance, scalability	Utilisation of edge computing in order to improve performance
(65)	Edge-based hybrid architecture with the hyperledger fabric and hyperledger ursa cryptography library	Can meet a real-time scenario in network latency	Scalability	More flexible access mechanisms
(54)	AI-based medical history summary generation with IPFS and Ethereum	Less time for studying old medical data	Performance issue while data size increases	Exploring a large-scale real-time application scenario
(62)	Random forest with ethereum framework for managing maternal health information	Better analysis to reduce pregnancy complication.	No real time data analysis.	IOT integration needed for better results.
(58)	IOTA tangle framework with masked authenticated messaging protocol	Performance, low latency	Trail count limited to 100	Real-time application scenario

#### 3.1.2 Supply chain management

**Figure 4:**
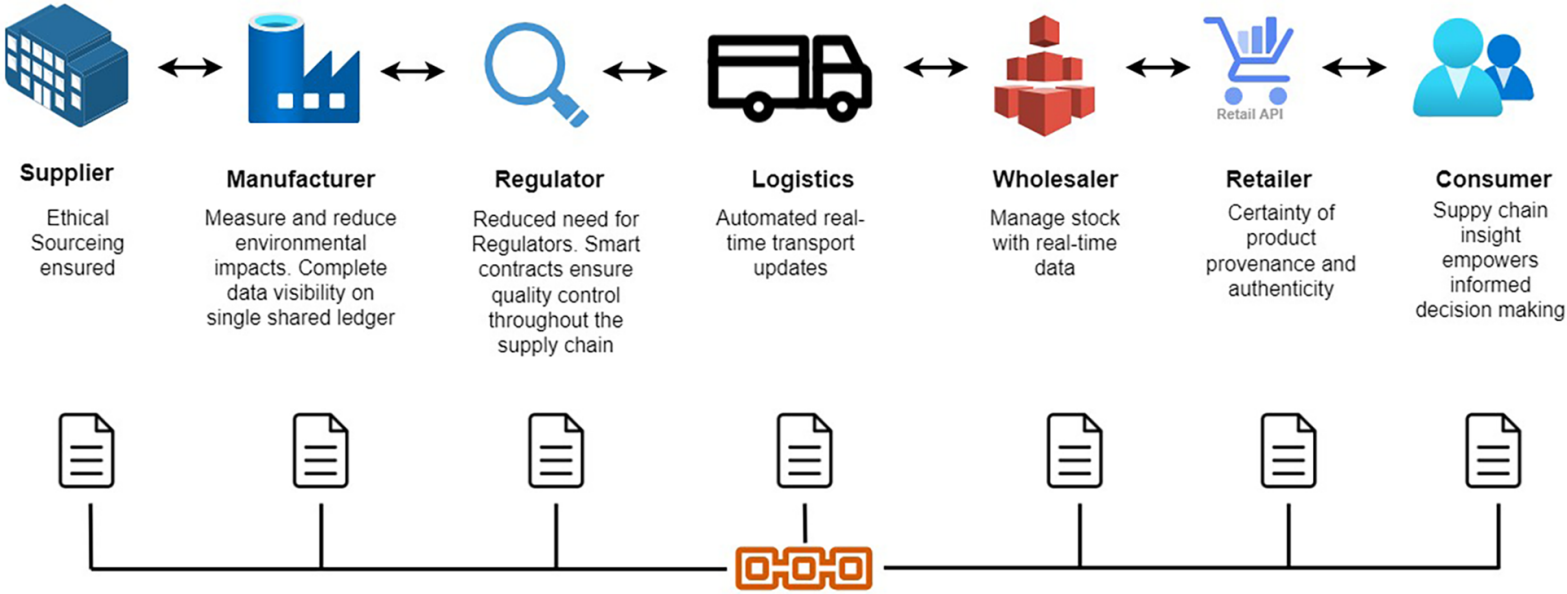
Supply chain management with blockchain.

It is a complex process in health care management because a lot of stakeholders are involved, and it has to be done in different stages, like from raw material gathering to delivering the product to the end user. So Maintaining efficiency, traceability, and transparency is a difficult task. But with BT inclusion, efficiency, traceability, and transparency can be easily achieved in supply chain management. Stakeholders can assure the authenticity and traceability of pharmaceuticals, medical devices, and other healthcare products. This would lower the prevalence of counterfeit items in the healthcare industry, which endangers patient safety. To achieve these benefits, BT-enabled supply chain management will record every detail of products at every stage, such as origin, manufacturing processes, quality control checks, transportation information, and more. BT supply chain management stakeholder interactions are shown in Figure 4. Blockchain technology is decentralized. Hence, this method reduces trust obstacles. Additionally, all participants have one reliable source. It also automates order placement via smart contracts, improving inventory management. Healthcare supply chain management includes drug tracking, medical device tracking, clinical trial supply chain management, and cold chain management.

**Drug traceability:** BT across the supply chain can track pharmaceutical drugs from production to distribution, preventing counterfeiting. Each medicine package is assigned a unique identifier that is recorded on the blockchain and corresponds to batch numbers, expiration dates, and production data. Cui et al. (66) presented a vaccine safety system using the FISCO BCOS consortium blockchain to trace vaccine supply from manufacture to customer. Jangir et al. (67) use the Ethereum blockchain for pharmaceutical supply chain management. They developed smart contracts for pharmaceutical raw material supply, manufacture, distribution, and retail.

**Medical device tracking:** With BT medical equipment life cycles, authenticity, quality, and compliance are simple to track. A tamper-proof ledger can record device creation, maintenance, and ownership transfers. The device’s path is visible to stakeholders. The methodology proposed by Omar et al. (68) improves the traceability of personal protective equipment (PPE). Full supply chain automation has been implemented with Ethereum smart contracts to build stakeholder confidence, transparency, and secure communication. Amin et al. (69) utilized the Hyperledger Fabric framework to enhance biomedical engineering supply chain security and transparency.

**Clinical trial supply chain:** BT integration helps to prevent the inclusion of counterfeit or unsafe medicine into trials, ensuring the safety and reliability of the materials utilized. It improves transparency and efficiency by tracking the movement of investigational drugs, biological samples, and trial-related papers, ensuring their integrity and regulatory compliance. It can also help to expedite interactions among many stakeholders, including sponsors, contract research organizations (CROs), and trial sites, allowing for more seamless coordination. Glover et al. (70) discussed blockchain use for improving efficiency, traceability, and patient safety in the clinical trial supply chain. They also discussed how BT will improve the authenticity of drugs and devices used in clinical trials by tracking their supply chains.

**Cold chain management:** It is vital to manage temperature-sensitive drug supply chains in the pharmaceutical and healthcare industries. Vaccines, biological samples, blood products, and perishable medications must be stored and delivered at precise temperatures to ensure effectiveness, safety, and quality. Maintaining optimal temperature conditions during transit and storage is critical in the healthcare business. BT integration provides an immutable record of temperature and surrounding conditions across the supply chain, ensuring compliance and product integrity.

Key aspects of cold chain management include temperature monitoring, temperature control, packaging, transportation, and documentation. Continuous temperature monitoring, aided by temperature sensors at storage facilities, vehicles, and delivery points, is critical. Data from these sensors is recorded and stored in a distributed ledger. According to the WHO, different medicines need to maintain a certain temperature range, like TT injections, which need to be maintained between $2{\;}^{\circ }$C and $8{\;}^{\circ }$C, and oral polio vaccines should maintain a temperature range of $-15{\;}^{\circ }$C to $-25{\;}^{\circ }$C. With the analysis of values stored in ledgers, smart contracts like programs can control the temperature by regulating the temperature of storage units such as refrigerators, freezers, and cold rooms. With this data, the supply chain can be automated for packaging by suggesting using insulation techniques like thermal insulation, gel packs, or dry ice packing. With these facilities, the transportation of temperature-sensitive products from manufacturing facilities to end-users is within the required temperature range. Distributed, tamper-proof documentation guarantees accountability and traceability at all stages.

Zhang et al. (71) proposed a framework with a private blockchain for maintaining temperature-sensitive products in the drug supply chain. Kim and Kim (72) proposed a framework for blood supply management systems from blood banks to hospitals with Hyperledger Fabric. According to regulations, whole blood and red blood cells should be stored between $+2{\;}^{\circ }$C and $+6{\;}^{\circ }$C, platelets and leukocytes between $+20{\;}^{\circ }$C and $+24{\;}^{\circ }$C, and plasma products below $-18{\;}^{\circ }$C. This complete framework and process have been automated with smart contracts. Additionally, technologies such as ML and AI have the potential to enhance the BT supply chain. Shah et al. (73) and Hu et al. (74) study the use of ML in the BT supply chain to assess medication safety and ensure timely distribution of medicines. Painuly et al. (75) and Long et al. (76) look into how AI is used to manage smart e-healthcare supply chains. An overview of supply chain management applications and frameworks with BT is presented in Table 3.

**Table 3:** Comparison of medical supply chain applications and frameworks with blockchain.

Paper	Implementation	Benefits	Issues	Further research
(66)	Drug tracing with the FISCO BCOS consortium blockchain	Security	Storage burden, performance	Suggested to try sharding
(67)	Drug tracing with ethereum private blockchain	Availability, transparency, privacy	Scalability, performance	Suggest to use ML algorithms to automate process
(68)	PPE medical device tracking with ethereum private blockchain	Secured data interchange, Transparency	No actual execution, like using ethereum test or mainnet	Automate supply chain process & Real time implementation
(69)	Biomedical supply chain with hyperledger fabric	Security, efficiency	Scalability not discussed	The comparison was completed with ethereum. Additional evaluation is required.
(77)	Personal blockchain	Transparency, traceability	Consistency, scalability	Practical implementation
(71)	Personal blockchain with cloud storage and deep learning	Automation of demand, Traceability	Cost analysis not done	Blockchain based features need to be addressed
(72)	Hyperledger fabric private blockchain	Cost efficient, transparency, efficiency	Disposal and storage errors not addressed	Practical implementation
(78)	Personal blockchain for counterfeit detection of COVID-19 vaccines	Transparency, efficiency, reduced cost	privacy, scalability	AI can used to detect fraudulent data

#### 3.1.3 Clinical trials

Clinical trials play a vital role in medical research because they enable the development of novel healthcare medical treatments. Various countries have strict laws managing clinical trials. In the United States, clinical trials are supervised by the Food and Drug Administration (FDA), which develops standards and rules. Clinical trials in the European Union are monitored by the European Medicines Agency (EMA). In India, the Central Drugs Standard Control Organization (CDSCO) monitors and supervises clinical trials. However, the International Conference on Harmonization (ICH) establishes globally recognized standards. These organizations and regulations guarantee that clinical trials follow Good Clinical Practice (GCP) guidelines, which include study permission, informed consent, reporting, and inspections.

**Figure 5:**
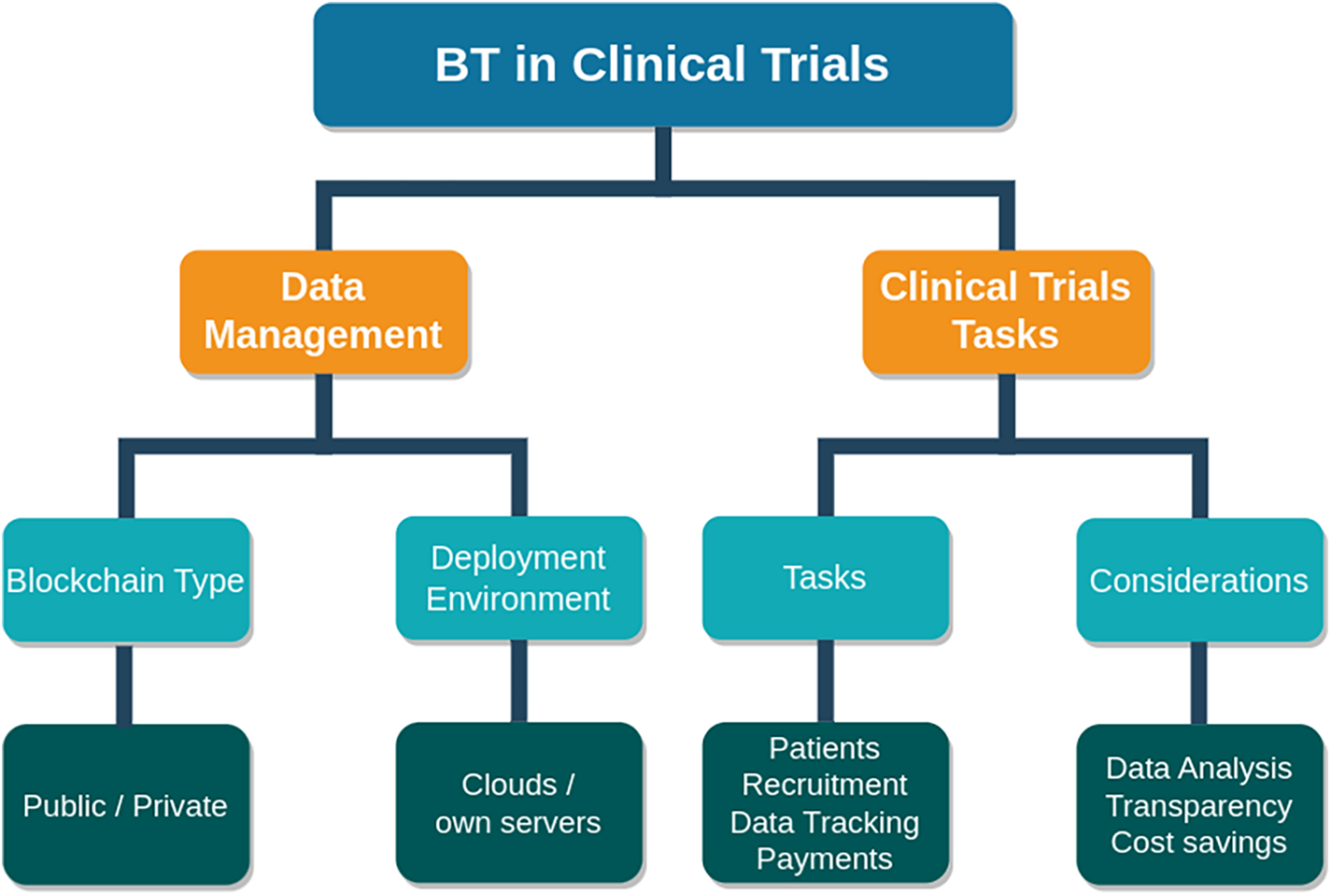
Clinical trials with blockchain.

Monitoring and validating these tasks are very difficult processes, and maintaining transparency and security for data is a crucial part. BT inclusion makes these tasks easy. Clinical studies can be more transparent and efficient with the help of BT. BT enables researchers to assure data accuracy, eliminate fraud, and streamline trial processes. Furthermore, blockchain can make clinical trials more affordable for patients while improving overall traceability and transparency by saving time and effort. Figure 5 depicts the stages of clinical trials with BT.

For example, Maslove et al. (79) and Nugent et al. (80) presented clinical trial frameworks. These are private blockchain environments developed with the Ethereum network using smart contracts. Can be accessed with their own web API. Patients and researchers need to register to participate in the clinical trials. Researchers will send requests to patients, and after request acceptance, patients will get incentives, and researchers will get data for analysis. Kuo et al. (81) introduced a QuorumChain to solve the site-unavailability problem in a hierarchical blockchain network for clinical trials. It helps to inherit the power of learning from a network of networks and increases learning continuity. Rajawat et al. (82) proposed a lightweight blockchain paradigm that assures data availability and integrity, which are critical for ML applications in clinical trial applications.

With BT inclusion in clinical trials like EHR management data, integrity and security will be increased. Smart contracts or similar programs can help automate data collection, consent management, and monitoring. Other than that, transparency will be provided by viewing the uneditable trial process, and traceability will be achieved. Most importantly, patient consent and identity information are protected, and automated payment is made for the collaborators. Finally, it provides seamless collaboration among stakeholders for the sharing of data, documents, and trial progress in a secure and efficient way. An overview of clinical trial applications and frameworks with BT is presented in Table 4.

**Table 4:** Comparison of clinical trial applications and frameworks with blockchain.

Paper	Implementation	Benefits	Issues	Further research
(79)	Data stores in databases and the metadata stored in Ethereum private blockchain	Data control, transparency, traceability, security	Cost barriers, limited stakeholders	More payment methods should be explored
(80)	Ethereum private blockchain and IPFS for large file storage	Transparency, credibility	Scalability	Needed to address scalability issue
(83)	Ethereum private blockchain and IPFS	Transparency, traceability	Scalability, interoperability	Real-time cost analysis is required
(84)	Hyperledger fabric with private channels	Privacy, security	High cost for deployment	Effective cost reduction required
(85)	Hyperledger fabric and compose with REDCap web client	Traceability, integrity	Scalability not mentioned	Recommended IOT integration
(86)	Ethereum private blockchain	Trial participants are selected automatically	No main network implementation	Real time analysis has to be done
(87)	Hyperledger fabric with Japanese cabinet sandbox regulatory	Availability, transaction throughput	Scalability not mentioned	Focused on single use cases need to focus on multiple
(88)	Ethereum based IOT data collection with ML for participants selection	Privacy, security	Performance metrics are not stated	Include further clinical study phases, such as auditing and monitoring.

#### 3.1.4 Health insurance claims

**Figure 6:**
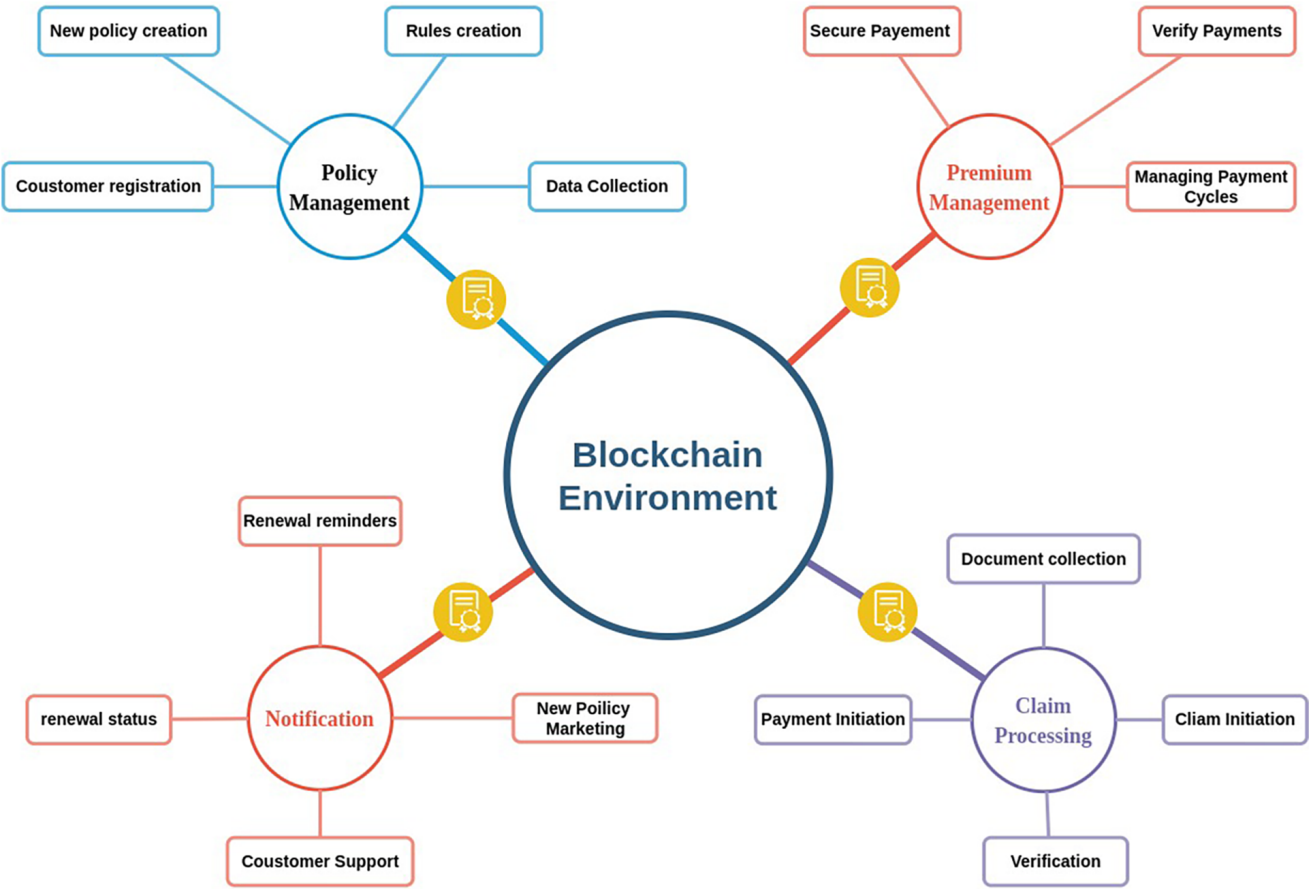
Insurance claims framework with blockchain.

Health insurance claims processing can benefit from BT. Stakeholders can verify patient information, prevent fraudulent claims, and increase trust and transparency all because of BT. BT also has the potential to expedite the claims process and cut down on administrative expenses. Information on health insurance policies that have been blockchain-enabled can be safely maintained on the decentralized ledger. This guarantees that everyone engaged has access to the most recent version of the policy details. When policyholders get more information about their coverage, their rates, and their ability to file a claim, everyone wins. BT can also be used to improve the claims processing system. By executing rules for claim validation based on specified conditions, like diagnosis codes or treatment procedures, smart contracts can automate the verification process. As a result, less time is spent manually processing claims, while quality and productivity are improved. BT also has important applications in detecting fraud. Claims of fraud can be uncovered more quickly and efficiently with the use of an immutable ledger that records all transactions. Blockchain’s distributed ledger design removes all potential centralized points of failure or manipulation. Figure 6 illustrates the health insurance framework with blockchain.

Zhou et al. (89) designed a blockchain-based storage system called MIStore to store and share insurance-related information for simple access in a safe environment. Sethi et al. (90) created a consortium blockchain, and Dhieb et al. (91) created a hyperledger-based blockchain framework to store medical data and used different ML algorithms like CNN to detect fraud reports for insurance claims. Dhieb et al. (92) proposed a smart insurance system based on blockchain and artificial intelligence for insurance automation. Overall, by incorporating BT into their operations, health insurers can better ensure the safety of their customers’ private information, such as medical records, from theft or tampering. An overview of health insurance claims applications and frameworks with BT is presented in Table 5.

**Table 5:** Comparison of Insurance management applications and frameworks with blockchain.

Paper	Implementation	Benefits	Issues	Further research
(89)	Smart contract based personal blockchain	Transparency, reduced cost, efficiency	Complexity, privacy	Use of an alternative blockchain system that is better suited for throughput
(93)	Personal blockchain	Fraud detection, security	Scalability not discussed	Further research needed to address scalability
(94)	IPFS based private ethereum blockchain	Security, efficiency	Complexity, privacy	Smart contracts improvement to improve privacy
(95)	Personal blockchain with hybrid consensus	Efficiency, transparency	Basic security provided	Need to add additional security measures
(96)	permissioned blockchain network	Transparency, accuracy, reduced cost	Interoperability, performance declines with more users	Interoperability, efficiency, and energy consumption
(97)	Personal blockchain framework to deal with existing transactions	Performance, transparency, automation	Under development	Scalability, integration of ML
(98)	Ethereum blockchain	Security	latency, Performance	Suggested to integrate lightweight node
(91)	PoA based hyperledger fabric blockchain with ML for fraud detection	Security, efficiency, accuracy	Complexity, scalability, privacy	Implementing new privacy rules and usage of additional ML algorithms.
(90)	Consortium blockchain with ML classifiers	Better fraud detection	Scalability not mentioned	Test with other algorithms.
(99)	Hyperledger fabric with custom parameters	Transparency	No encryption, Performance is less with large dataset	Encryption of data with fine-grained access control

### 3.2 Blockchain impact in the healthcare industry

BT’s increased security protects patient data from theft or hacking attempts, allowing patients to select who has access to their medical records, hence increasing privacy and information protection. EHR management is simplified with BT, giving doctors access to patients’ complete and accurate medical records, thereby improving care delivery. Furthermore, blockchain can increase healthcare transparency by acting as an immutable ledger that records all transactions, potentially improving patient care, accountability, and waste reduction.

Due to these advancements,leading organizations in the healthcare sector are rapidly demonstrating the transformative potential of blockchain technology by deploying cutting-edge solutions. Companies like IBM have developed blockchain technologies for the healthcare sector, enabling secure and interoperable data transmission among stakeholders. Startups like Medicalchain are using blockchain technology to put patients in charge of their medical records and give them the option of sharing only the information they deem necessary with their doctors. Blockchain has been utilized by leading healthcare technology company Change Healthcare to improve billing and claim processing by decreasing manual work and human error. Companies like these, and many more like them, demonstrate the real-world utility of blockchain in healthcare by providing solutions to pressing issues like data privacy, security, and efficiency in a sector that is developing at a breakneck pace. An overview of these companies has been presented in Table 6.

**Table 6:** Real world applications with blockchain in healthcare.

Company/Country	Implementation	Description
Patientory (100)	Health record sharing with ethereum blockchain	The aim is to reduce the repeated tests. Patients and the health industry alike can utilise this application. Payments are done with PTOY tokens.
Medicalchain (101)	Health record sharing with hyperledger fabric	Third parties obtain and interact with EHRs through the API with the user’s permission. uses MedToken.
MediBloc (102)	Health record sharing with panacea blockchain	Incorporates the PBFT algorithm for delegated proof of stake consensus. Uses MED coins.
EncrypGen (103)	Genetic data sharing for clinical trials with ethereum blockchain	Control who can see your genomic information and how much you can charge for access to it. It has its own token system.
Avaneer (104)	Clinical trials with IBM blockchain	Can share any health data for clinical trials. The main objective is to increase productivity and openness.
Chronicled (105)	Supply chain management with private blockchain	Developed a private blockchain called mediledger to track the drugs and medical devices. Mainly focused on cold chain management.
Skuchain (106)	Supply chain with EC3 blockchain technology	It provides a framework to work with different platforms like Bitcoin and Ethereum-based drug supply chains.
Aetna (107)	Clinical trials management with IBM based blockchain	Together, Aetna and IBM tested a blockchain based approach to the health insurance process that should save costs and promote transparency.
Estonia (108)	Healthcare, e-resident programme with KSI blockchain technology	Estonian started testing BT in 2008. The estonian information systems authority will monitor blockchain networks.

Numerous studies have been conducted to examine the impact of BT on the healthcare sector. We reviewed notable studies and summarized their key details in Table 7, providing insights into their methods, strengths, and areas for future research. In our review of the current literature, we discovered important research gaps in the knowledge and characterization of the issues related to BT in healthcare. Specifically, challenges have not been properly separated into technical and non-technical categories. Furthermore, we found a lack of precise descriptions of solutions, particularly when distinguishing between on-chain and off-chain techniques. To fill these gaps, our study categorizes difficulties as technical or non-technical and gives a thorough evaluation of solutions, distinguishing between on-chain and off-chain approaches. Furthermore, we structured these findings into various levels within the blockchain architecture to improve clarity and application in tackling healthcare-specific issues.

**Table 7:** Summary of eligible studies.

Paper	Approach	Strengths	Further research
(109)	Review of blockchain applications in the health care sector.	Discussed Healthcare integration with BT.	scalability, latency, and costs need to be addressed.
(110)	Review of BT integration in different healthcare use cases.	Explained how BT used to overcome traditional issues In healthcare.	Performance, scalability, security, and privacy issues to be addressed.
(111)	Review of BT integration in different healthcare use cases.	Investigated different BT based healthcare including applications of private organizations.	Performance, scalability, security, and privacy issues to be addressed.
(112)	Review of security flaws with respect to healthcare applications.	Methods for mitigating security problems have been highlighted.	Performance issues and solutions for issues to be addressed.
(113)	A review of BT usage in healthcare	Standardization, latency, and scalability issues were discussed.	Solutions for issues need to be addressed.
(114)	A review of Blockchain applications in healthcare.	Performance, scalability and computing power problems discussed.	Solutions for issues need to be addressed in detail.
(115)	Review of BT integration in different healthcare use cases.	An overview of BT in healthcare and its issues are presented.	Detailed investigation on scalability, security, and privacy solutions.
(116)	Review of the possibility of BT in healthcare.	Presented use cases and issues with integration.	Detailed investigation on scalability, security, and privacy solutions.
(117)	Review on scalability issues in blockchain healthcare applications.	Architecture and data optimization on-chain solutions are explained.	Optimization of smart contracts and off-chain solutions to be discussed.
(118)	Investigation of key issues in healthcare data integrity	Issues are discussed with few solutions.	Detailed explanation of the solutions is required.
(119)	Review of blockchain usage to handle the COVID-19 pandemic.	Detailed explanation of BT usage for pandemic is given.	Performance issues and solutions for issues to be addressed.
(120)	Review of BT based initiatives that attempted to maintain privacy and security in healthcare.	The benefits and negatives of using BT in healthcare vs. traditional approaches were discussed.	Performance issues and solutions for issues to be addressed.
(121)	Review of healthcare data quality concerns with blockchain technology.	Challenges like technological, adoption, and operation are discussed.	Solutions for to overcome these challenges need to discuss.

## 4 Issues related to blockchain in healthcare

Regardless of the potential benefits of BT for healthcare data management, some issues must be addressed. For example, due to decentralized nature every participant in the network can access the data stored on the blockchain. This data is secured with encryption techniques but it is very difficult to maintain the data privacy and confidentiality. Furthermore, the immutability of blockchain systems may provide challenges in the event of data breaches or errors, as inaccurate data might be difficult to modify or erase once it has been posted to the blockchain. Further research is needed to address these challenges and develop robust blockchain-based health record management systems that can meet the complex needs of the healthcare industry. Most of the healthcare application has been developed with the public network Ethereum. So this network drawback shows effect on healthcare applications. Here we discuss the issues rising in blockchain healthcare.

### 4.1 Technical or performance issues

#### 4.1.1 Storage maintenance

BT is developed to combine the list of transaction information and bind it to a block, which requires low storage ability. But in healthcare, Medical records, test reports, MRI and X-ray images, and other forms of graphical data are only some examples of the massive amounts of data that need regular processing. As the size of the blockchain grows, maintaining and replicating the full blockchain across all network nodes becomes more complex, resulting in increasing storage demands and decreasing performance.

Especially Ethereum (11) and Filecoin (122), like networks, will charge more for storing images. For instance, the cost of storing 256 bits of data on Ethereum is 20,000 gas points. Other networks like Hyperledger (29) and IOTA Tangle (36) offer no-cost image storage facilities. Even though maintaining complete data with images at every node becomes very costly, it is very concerning, especially for smaller firms or resource-constrained healthcare providers.

#### 4.1.2 Security

In traditional systems, BT has removed one of the biggest threats to the security and privacy of the information by preventing third parties from doing the transaction. However, the decentralized structure of BT raises new security concerns. Even though all nodes can see all the information on the network, accessing data requires permissions. Without external management, patients may have to name several agents with access to their medical records. In times of patient emergencies, it can be difficult to allocate permits. Researchers like Rajput et al. (123) and Hasavari et al. (124) have presented solutions to give access for practitioners to patient data without requiring explicit patient agreement to do so, which could potentially mitigate this problem. A careful consideration of the balance between data security and patient care may be necessary, as this approach allows doctors to access a patient’s medical history for better treatment decisions but also raises concerns about data security as it involves the sharing of patient information without direct patient consent.

Another scenario for security concerns is the use of off-chain storage like IPFS and clouds to overcome the storage maintenance burden for BT healthcare applications. The Ethereum-based healthcare models proposed in (54, 63, 80, 83, 94) all use IPFS for image storage, while the Ethereum-based healthcare model proposed in (64), the Hyperledger Fabric-based model proposed in (50), and the personal blockchain-based model proposed in (71) use cloud storage. In these models, images are stored outside of the chain, so security is compromised. Qaqish et al. discussed the security issues related to cloud-based blockchain off-chain storage prospectively (125).

#### 4.1.3 Throughput

The low transaction throughput is another major drawback of blockchain networks. This problem refers to the limitation on the number of transactions a blockchain network can complete in a particular time frame. Basically, this problem arises on platforms that use PoW consensus mechanisms, such as traditional public blockchains like Bitcoin and Ethereum. In a BT-decentralized environment, several nodes evaluate and verify transactions, which may result in slower transaction processing speeds when compared to traditional centralized systems. With drawbacks, transaction delays, and network congestion when dealing with a high number of healthcare transactions at the same time will become difficult. Most of the Ethereum-based models exploit this issue because they use the POW algorithm. Other than this, other blockchain platforms also use it for large-scale applications, like the Hyperledger fabric-based proposed model in (99) and personal blockchain-based models proposed in (96).

#### 4.1.4 Scalability

BT can be slow and inefficient when it is used to store large amounts of data. This could be a problem for EHR systems, which typically contain a large amount of patient data. It emerged as a key barrier to the adoption of BT in healthcare applications. The low transaction throughput of blockchain networks is a major scalability concern. Another factor influencing scalability is the growing storage requirements of blockchain systems. Furthermore, blockchain consensus procedures such as proof-of-work or proof-of-stake might exacerbate scalability issues. These strategies necessitate network participants doing computationally expensive activities or staking large sums of cryptocurrency, making the validation and verification process time-consuming and resource-heavy. The computing needs and energy consumption associated with reaching consensus may become unsustainable as the network scales.

Scalability issues in healthcare models can be partially resolved by implementing off-chain computing. One approach is using off-chain computing, like the Ethereum-based healthcare models proposed in (63, 67, 68, 80, 83) which all use IPFS for image storage. This method eliminates decentralisation advantages, which introduces security concerns. To overcome this, additional security measures have to be implemented. It leads to increased complexity and decreased performance. Due to this, the scalability of the application will be affected. The other approach is using a customised blockchain network instead of public networks. Hyperledger Fabric-based models proposed in (65, 69, 87), and the personal blockchain-based models proposed in (77, 78, 97) have been aimed at overcoming public network problems like Ethereum off-chain storage. However, these frameworks also exhibit scalability issues due to the complexity and maintenance of the applications.

### 4.2 Non-technical issues

#### 4.2.1 Adjustment issues

Since BT is implemented with an eye on genuine progress in the healthcare sector that may be challenged by adaptation. There could be a need for verified as well as strict international standards. The already defined standards may be helpful to compute the dimension and set up the data interchanged in blockchain services. Such standards may not only examine the shared information but also remain protective measures. For instance, a healthcare provider that has used traditional medical record management systems for decades Moving to blockchain-based EHRs is a big change. Doctors, nurses, and administrative staff may need training to use the new computerised system. Standardising data formats and protocols for interoperability with other healthcare providers and systems can be difficult. New processes and rules may be needed to secure and protect patient data on the blockchain.

#### 4.2.2 Social issues

BT is developing and faces social concerns like social transformation and technical issues. Technology that differs from normal research methods may be difficult to achieve. The healthcare industry is making progress towards digital data, but the barrier to completely transforming the technology, especially blockchain, has yet to be established. The medical industry’s slow acceptability of technology and guidelines make it difficult to encourage medical specialists to migrate from research data to blockchain at the right time. For instance, medical researchers are used to leveraging traditional data collection and processing methods. They trust these tactics over time. Blockchain technology for secure and transparent data sharing and collaboration may be met with opposition due to its perceived complexity. Researchers may resist blockchain-based data management due to social pushback. Convincing them to utilise blockchain entails showing its benefits, addressing data security issues, and delivering user-friendly tools and interfaces.

#### 4.2.3 Lack of standards

The use of BT in EHR administration is not yet standardized. This can make it challenging for several healthcare organizations to work together. This procedure calls for cautious thought, teamwork, and compliance with all applicable regulations. For instance, two healthcare organizations are independently deploying blockchain-based EHRs. Both hospitals choose their own data format and encryption. Lack of standardized data formats and interoperability protocols makes sharing and integrating medical records between hospitals difficult. Patients may require several tests, and doctors may have difficulty gathering critical information. Healthcare organizations struggle to share medical data due to this lack of standards. The DAO (Decentralized Autonomous Organization) hack (126) happened in 2016. It is a smart contract-based, decentralized financial management system for organizations. It is based on the Ethereum blockchain platform and automates financial transactions on a voting basis. Organization ownership and voting power on a blockchain using tokens or cryptocurrencies Initially, it works perfectly and gains popularity. In a short time, 12.7 million ETH invested in the DAO smart contract. However, due to a technical error in the smart contract code, a hacker was able to steal 3.6 million ETH in a few hours. This flaw in the smart contract could not be fixed because of the immutable nature of blockchain technology. As a result of this event, many people in the Ethereum community began debating the possibility of reversing the DAO contract’s transactions by making code changes to the smart contract. However, this suggestion was met with rejection from some members of the community who were passionate about maintaining blockchain basics and accepted the hack. The argument progressed into a controversial hard fork, which split the Ethereum network in half. One group, known as Ethereum Classic, modified the smart contract and rolled back the transactions after the hack, while another group stuck with the immutability of the blockchain and accepted the consequences of the breach. Social acceptance, governance concerns, and the necessity for standards within the blockchain community are all brought into question when a hard fork is implemented. In the event of a hard fork, all users can potentially double their cryptocurrency holdings by taking part in both chains that emerge. While this creates an economic opportunity, it also highlights the need for community acceptance, ethical judgments, and the establishment of transparent rules for managing blockchain. In another instance, the Party Wallet hack (127) happened in 2017 and allowed hackers to steal 150,000 ETH. It also leads to standard and adjustment challenges. The biggest adjustment challenge for users is forgetting the blockchain wallet keys (128). If a user forgets the wallet keys, unlike traditional systems, there is no way to recover them. As a result of this, a Bitcoin user lost control of 7,002 bitcoins in 2021.Despite these challenges, BT is a promising new technology with the potential to make a significant impact on the healthcare industry. As the technology matures and the challenges are addressed, BT is likely to play an increasingly important role in EHR management in the future.

## 5 Proposed solutions for performance issues

**Figure 7:**
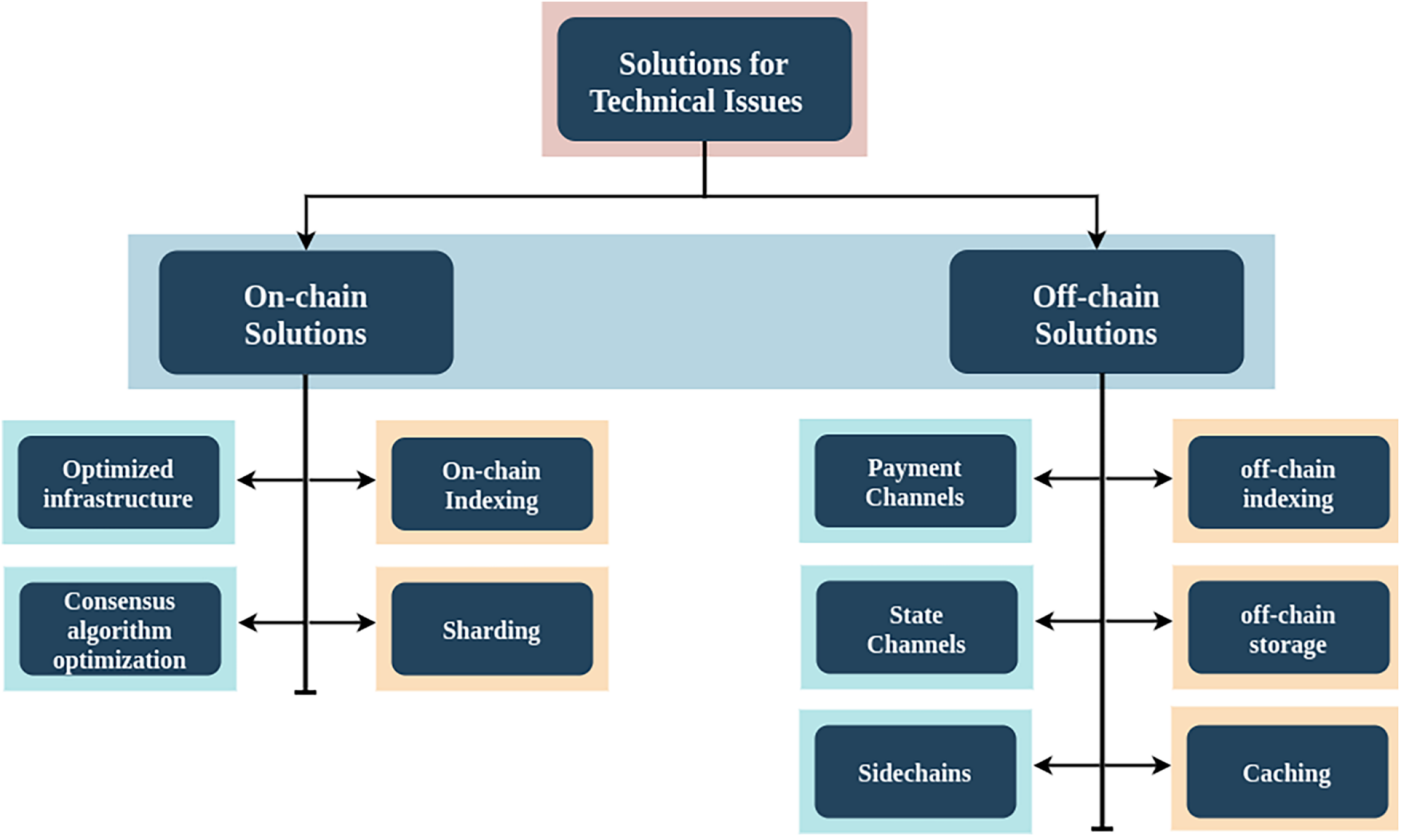
Solutions for technical issues for blockchain.

As mentioned in Figure 7, there are several solutions proposed to overcome blockchain performance issues on both off-chain and on-chain, like sharding, sidechains, on-chain indexing, off-chain indexing, optimised infrastructure, consensus algorithm optimisation, caching, off-chain storage, and off-chain transaction processing through payment channels and state channels. For non-technical issues, there are no fixed or proposed solutions available. But these non-technical issues can be overcome with the help of practices like creating more awareness regarding blockchain, increasing stakeholder engagement and collaboration, creating regulatory frameworks and standard development procedures, creating more proof of concept, and continuous monitoring and evaluation. In this section, we will discuss solutions proposed for technical issues in BT for healthcare.

### 5.1 On-chain solutions

These solutions refer to techniques or functionalities that occur directly on the blockchain network itself. These solutions mainly depend on modifying the underlying blockchain infrastructure, which involves changes in transaction storing, processing, and validating processes on the blockchain. This can include processes like transaction accessibility on-chain indexing, transaction or data storage sharding, optimised node infrastructure to improve data Organization and access, consensus algorithm enhancements, and privacy and confidentiality solutions like zero-knowledge proofs. These techniques can enhance the performance and efficiency of blockchain.

#### 5.1.1 On-chain indexing

Notability It is the initial approach to improving transaction access time. In the Ethereum blockchain, the Merkle Patricia Trie (MPT) data structure plays a crucial role for indexing (129, 130). MPT stores the states of each account within the blockchain, including its balance, contract code, storage, and other account-specific information. It is a variation of the Merkle Tree data structure, specially designed to efficiently store and retrieve key and value pairs in a secure and tamper-proof manner. MPT organises data in a hierarchical structure. As users increase, it gives poor performance while searching for specific transactions and retrieving them. Another drawback of MPT is that tracking and analysing real-time changes is not possible.

Events are another core part of smart contracts. Because in the Ethereum blockchain every transaction receipt is stored in event log entries (logs), events store the real-time updates of particular smart contracts, and this event data can be indexed (131, 132) and acts as a checkpoint in Ethereum Virtual Machine (EVM) code execution. With this, efficient querying will be possible because there is limited data to be searched. Another potential method is contract metadata indexing. It is one of the subparts of the event indexing mechanism. This index creates metadata related to smart contracts, such as their ABI (application binary interface) and source code. But in practice, storing and querying based on smart contracts is a difficult option when it comes to large data sets.

In the meantime, researchers adopted a linked list data structure for indexing the data. This technique uses a linked list to permanently store the final transaction hash alongside the original transaction data. In order to complete a query, it must first complete the header transaction. This method has high performance compared to smart contract querying. However, the query efficiency may be quite low because of the chain structure. Researchers experimented with various methods in response to this approach. One such method is the creation of static indexing (133) for the transactions. It is achieved by embedding the first 2i transaction timestamp and hash values into the current transaction. Another method is a hybrid index mechanism called EtherH, which has been created with the help of B-Tree and skip lists (134) on the Ethereum blockchain. Overall, these indexing techniques have improved efficiency, and because they are built as on-chain solutions, they are quite secure. This has certain disadvantages as an on-chain method, such as the need for a large amount of extra storage space, which raises the cost of maintaining a node on the blockchain. increased scalability and complexity in the application architecture.

Indexing techniques applied to healthcare blockchain applications can improve data management and transaction access, improving healthcare systems overall. Healthcare blockchain solutions’ efficient indexing and optimised data structures enable faster patient record access, simpler claims processing, better pharmaceutical traceability, and better event monitoring. These strategies provide the safe and effective management of essential healthcare data, improving healthcare services.

#### 5.1.2 Consensus algorithms

The consensus algorithm is one of the necessary components of the blockchain. It will ensure agreement and trust among distributed nodes. After indexing techniques, developing new consensus algorithms is a popular concept for blockchain researchers. The primary blockchains, Bitcoin and Ethereum, developed their environments with the use of the POW consensus algorithm. But according to the study (135), Bitcoin mining has the potential to add $2{\;}^{\circ }$C to global warming. Because POW requires more energy to do the work, To overcome this, several researchers developed numerous energy-efficient consensus algorithms. For instance, researchers from Cornell University proposed Bitcoin-NG in 2015. It is a modified version of the traditional POW consensus algorithm. A modified POW approach gives every winner the facility to create several new blocks instead of one block (136). Other than this, several other algorithms have been proposed, like POS, PBFT, and PoET (137). Among these algorithms, POS is the most popularly adopted.

Ethereum updated version 2.0 (Eth2) is using the POS algorithm over the initial version (Eth1) POW algorithm. In POS, for the creation of the next block set of validators, take turns and take a vote. This vote weight depends on the size of its stake deposit. PoS has the benefits of energy efficiency, security, and reducing the risk of centralization. Initially, Quantum Mechanics and other researchers supported the idea of replacing POW with the POS concept in the Bitcoin forum. But Kiayias (138) was the one who first used it to develop new blockchain protocols. Ethereum planned to roll out Eth2 phase-wise. As an initial phase, it was planned to replace POW with POS, and it was released as The Beacon Chain on 1 December 2020 (139). Before implementing proof-of-stake on the Ethereum Mainnet, it was built to make sure the underlying logic was robust. In the next phase, the merge upgrade is performed on the beacon chain. This phase merged the Beacon chain with the Ethereum Mainnet on 15 September 2022. Now, beacon chain POS acts as a consensus layer for the main Ethereum mainnet. Other than Eth2, Cardano, Cosmos, and Peercoin are some other platforms that use POW.

PBFT is another popular algorithm used for private blockchain networks. In this, a predefined validator group has been created, and then validators will communicate and agree on the data. With this feature, it gives fast throughput and comes with quick agreement on data. Corda, Hyperledger Fabric, and Quorum blockchain platforms use PBFT. The Intel-created PoET consensus algorithm uses a trusted execution environment (TEE) (140) to achieve consensus in a distributed fashion. The Hyperledger Sawtooth platform uses the PoET consensus algorithm. Several other algorithms are also available, but they are all easy to use when you create a new blockchain network, but for already existing networks, the adoption of another algorithm takes more time and is a very difficult task.

In conclusion, consensus algorithms play a pivotal role in shaping both blockchain technology and healthcare. Energy-efficient consensus algorithms, as demonstrated in the paper (25). They enhance scalability, reduce costs, and ensure data integrity. In another proposal (57), EHR management is proposed with a delegated Byzantine Fault Tolerance (dBFT) algorithm to achieve scalability. Future healthcare apps built on the Ethereum platform are also expected to greatly benefit from the switch to POS in version 2.0. As an outcome, better consensus algorithms will lead to safer healthcare data management, smoother processes, and higher-quality care for patients.

#### 5.1.3 Sharding

It involves breaking up the blockchain into smaller partitions called shards. In this method, a complete blockchain network is divided into smaller subnetworks, and each subnetwork has its own set of nodes and operates independently for storing and processing the data related to the subnet in the blockchain. This can improve transaction access time by distributing the workload across multiple nodes. Another approach is to store the transaction data in parts. These parts are created based on either transaction data or the state of the blockchain (141). Initially, Ethereum planned to adopt sharding techniques for its network due to network congestion. After the merger of the Beacon chain and Ethereum Mainnet, Eth2 is planned to roll out sharding mechanisms in the next phase (142). Ethereum has been working on implementing Danksharding instead of sharding for its real-time applications (143). Overall, due to its distributed workload, sharding can enhance the scalability of blockchain networks by accommodating more participants and can increase performance by enabling a larger number of transactions to be processed per second. With these features, it’s developed into a promising technology with the potential to completely change the blockchain sector. However, it is still a relatively new technology, and before it can be broadly embraced, it must first overcome a number of obstacles. Potential drawbacks of this technique are loss of security because each shard has a smaller number of nodes and is therefore more vulnerable to attacks, like the risk of malicious users taking control of a shard. and it is a complex technique to implement. To implement this technique, it requires adopting new consensus algorithms because all sheds should come to a single agreement for the final version of the complete blockchain and all shards should communicate properly.

For instance, Omran et al. (144) proposed a sharding-based healthcare application and concluded that scalability and performance are achievable. Other works by Abbas et al. (145) and Hafid et al. (146) on simulation showed that the security concerns were substantial, despite the application’s scalability and performance.

#### 5.1.4 Optimised infrastructure

Optimising the infrastructure of the blockchain can also improve transaction access time. It involves modifying the current blockchain architecture and implementing new consensus mechanisms. An effective network architecture is one of the methods that can accommodate both present and future business demands. Scalable, dependable, and secure are its main features. Another method is the node optimization technique. It is the process of configuring and managing nodes in a way that maximises performance and minimizes costs. This can be done by optimising the placement of nodes, the configuration of network settings, and the use of resources. Network optimisation performs by using faster hardware, optimising software, and minimising network latency. The benefits of infrastructure optimisations are improved performance and increased scalability. Drawbacks include increased hardware costs and increased maintenance requirements. Mainly, you have to create a new blockchain network and deploy it. Integrating with existing platforms is a very difficult process, and failure chances are very high, especially for public blockchains.

Several researchers have proposed solutions by adopting architecture optimization. In node optimization, instead of using traditional peer-to-peer networks, researchers used clustering mechanisms. In this group of nodes created as a network, only group heads will receive the data and distribute it within the subnetwork instead of distributing it to every node. It has been presented in different ways, like tree structure clustering (147), DHT clustering (148), and geographical proximity sensing clustering with the help of the K-means algorithm (149). In network optimisation, sharding can be used. The final method is optimising the block structure or size. In this method, instead of using the traditional block structure, a modified block structure will be used, which can decrease the bandwidth consumption of the network (150). Another method is increasing the size of the block, which allows more transactions in one block. Many well-known blockchain platforms have done blockchain architecture optimisation, like Ethereum, using a technique called Danksharding to divide the network into smaller subnetworks as a network optimisation, which can help to improve performance and scalability in its new version, Eth2 (143). The Bitcoin network adopted block optimisation by increasing the size of the block on a few occasions (151).

Bitcoin XT, announced in 2015, features an 8 megabyte (MB) block size instead of the traditional 1 MB. It was released as a soft fork of the main Bitcoin network. It works well at first, but then presents problems. Rapid escalation in block size created new security issues, and larger blocks may not be confirmed every 10 min, causing delayed confirmation periods. After it halted, Bitcoin Classic was forked from the main Bitcoin network in 2016 with 2 MB blocks. Not a success either. Bitcoin Cash is the successful version. It was hard forked in 2017 with 32 MB. Instead of forking existing networks and facing difficulties, one can create their own customised private blockchain according to requirements with the help of the HyperLedger Fabric Framework.

Scalability, dependability, and security are the hallmarks of an optimised network architecture that is effective in meeting current and future healthcare needs. Node optimization approaches include clustering mechanisms to increase the efficacy of data distribution in healthcare sub networks by decreasing redundancy and latency. Sharding and other network optimisation techniques can increase the capacity of healthcare blockchain networks. Moreover, by optimising block architectures, such as by adjusting block sizes, healthcare data transactions can use less bandwidth.

### 5.2 Off-chain solutions

Involves moving some of the computation and data storage off of the main blockchain onto an external platform or layer. In this Layer 2 solutions are created that act as intermediaries between users and the main blockchain. With the Layer 2 solutions, there is no need to modify the current working blockchain network. Some techniques related to this are payment channels, state channels, off-chain indexing, caching, and sidechains. This additional layer can improve transaction access time by reducing the load on the blockchain and allowing faster processing. Reduced processing and storage demands on the main blockchain are another way in which these solutions boost scalability.

#### 5.2.1 Payment channels

In the context of blockchain technology Payment channels were one of the earliest off-chain solutions introduced for improving scalability and performance. It mainly focuses on facilitating fast and efficient payment transactions off-chain. It works as a bidirectional channel between two or more participants. Payment channels allow for the transfer of digital assets without recording each transaction on the main blockchain. With this approach, it has expertise for handling high-frequency and small-value transactions, making it well-suited for use cases such as micropayments, streaming payments, and recurring payments. Payment channels provide several advantages over on-chain transactions. They offer instant transaction confirmations, as the parties involved can quickly update the channel’s balance without requiring confirmation from the blockchain. This real-time settlement capability enables fast and seamless peer-to-peer transactions. Furthermore, payment channels significantly reduce transaction costs. Since most transactions occur off-chain, participants can avoid the fees associated with on-chain transactions. This feature makes payment channels economically viable for small-value transactions and use cases that require frequent transfers. The Lightning Network, initially proposed in the whitepaper by Joseph Poon and Thaddeus Dryja, introduced payment channels on the Bitcoin blockchain (152). For the Ethereum blockchain, Raiden Network proposed a facility using payment channels (153). In another research study by Lind et al. (140), they proposed a Teechain blockchain framework with the TEE Consensus Protocol. Payment channels in healthcare enable fast, accurate transactions, reducing administrative costs and errors.

#### 5.2.2 State channels

State channels are suitable for off-chain transactions and work like Payment Channels. Payment channels just enable payments, but State Channels allow data change. Hence, they work best for Ethereum-like networks. A few peers can create a secured personal private channel to conduct an arbitrary number of off-chain transactions, like exchanging medical data and communication, by submitting only two on-chain transactions to open and close the channel. This method bundles a few transactions and maintains their transaction hash in the main chain instead of all transactions. Only channel peers can access these transactions. It reduces network congestion, the main blockchain burden, and increases scalability and accessibility. It allows high transaction throughput, lowering user expenses. Possible cons are reduced decentralisation, security risks, and complexity. Since fraudulent or malevolent channel behaviour could cost payments, it requires confidence between participants. State channel capacity is also constrained by initial funding deposits. Raiden Network proposed a facility for the Ethereum network by using state channels for data transfer and payment channels for quick payments (153). Another solution proposed by Podgorelec et al. (154) clarifies that state channels increase performance.

With state channels in blockchain technology, It could transform healthcare. State channels enable real-time, off-chain interactions while ensuring blockchain security and openness. This allows quick, cost-effective patient-doctor interactions, medical data access, and insurance claim processing in healthcare. Healthcare is more accessible and convenient since patients can get services and consultations faster.

#### 5.2.3 Off-chain indexing

Off-chain indexing refers to the practice of keeping track of a database index separate from the blockchain itself. The indexes of on-chain data will be stored in a separate database. This can include things like transaction history, user accounts, smart contract events, etc., all of which are indexed off-chain by specialised nodes known as indexers. This approach helps to reduce the amount of data that needs to be stored on the chain, which in turn can improve its performance. This approach has been proposed to overcome the drawbacks of the on-chain indexing mechanism. Its main aim is to facilitate users’ ability to search for and retrieve relevant information quickly from large amounts of data. SQL indexing is a tried-and-true method for organising data in traditional databases (155). Another type of indexing mechanism for off-chain indexing is the use of noSQL databases like MongoDB, and graph indexing technologies have been used (156). Researchers created a project called Bitcoin Database Generator (157) with the use of sql tables and Bitcluster (158) with the use of noSQL database MongoDB and made it freely available for other researchers at github. Potential drawbacks of this approach include centralization because off-chain data will not be stored as blockchain-distributed. Maintaining consistency for off-chain data related to on-chain data requires a good method. And mainly rises new security risks like unauthorised access and data leakage.

An investigation done in papers (88, 91) raises concerns when applying the ML technique to blockade-based large amounts of medical data. Off-chain indexing can overcome this by lowering data access costs and improving operations. Study done by Tanwar et al. (159) on ML adoption in blockchain applications supports this argument. Their study suggested database integration for Blockchain application performance improvement. Healthcare providers can retrieve patient information, medical histories, and clinical data faster by keeping comprehensive data off-chain and optimising data indexes. Patient care and treatment outcomes improve as healthcare providers make faster, data-driven decisions. Off-chain indexing also allows many institutions to collaborate on research and analysis without disclosing sensitive patient data. This accelerates medical research and innovation while protecting data and complying with regulations. Off-chain indexing allows healthcare practitioners and researchers to maximise data use while reducing access obstacles and protecting patient privacy.

#### 5.2.4 Caching

Frequent transaction data is stored in order to retrieve it quickly without querying the blockchain. This can greatly minimize common data transaction access time. Blockchain applications use caching by temporarily storing data in a fast cache like RAM so it can be retrieved and reused without needing to use hard drives. Caching may cause data discrepancies since cached data may not be up-to-date with the blockchain. Wang et al. (160) discussed this method. Because it stores previously searched queries, this technique may store more data and be inconsistent. Blockchain applications can employ several caching methods, depending on the use case. In client-side caching, clients or user interfaces cache frequently accessed data. DApp users experience less latency and better responsiveness. In node-level caching, nodes running on the same machine as the application store frequently access data to make it available faster. And in distributed caching, distributing cached data across application network nodes improves load balancing and system performance.

Heo et al. (161) proposed a multi-level distributed caching framework. It stores the frequently accessed information at nodes and distributes it across the chain. Yamanaka et al. (162) proposed a client-based catching mechanism for IOT-based healthcare applications. Similarly, it can be applied in healthcare to minimize on-chain operation load by storing frequently searched insensitive data like hospital and doctor details.

#### 5.2.5 Sidechain

Sidechains have become one of the most used mechanisms in recent trends and have become a well-known off-chain option to solve scalability, interoperability, and specialised use case requirements. In this method, separate blockchains can be created to work side-by-side with the primary blockchain. It allows users to conduct transactions without burdening the main chain. Throughput for transactions is enhanced, and special functionality is made possible by allowing users to transfer assets between the main blockchain and the sidechain.

With a focus on Bitcoin performance, Adam Back et al. (163) first proposed the sidechain concept, which is compatible with other blockchain networks. This allowed the creation of unique features and applications not available on the primary chain. Developers can try new consensus, scalability, privacy, and smart contract solutions due to this flexibility. Another notable implementation of Sidechain is the Liquid Network (164). It is developed as a federated sidechain by Blockstream that utilises the Bitcoin blockchain. Plasma Framework (165) proposes a hierarchical sidechain anchored into the main contract put onto Ethereum Main-Chain as a child chain. In the healthcare sector, Donawa et al. (166) suggested a side chain structure for healthcare that is based on a personal blockchain that uses the POA consensus algorithm. The primary goal is compatibility with the Ethereum infrastructure. In another research study, Hirtan et al. (167) proposed a side chain framework. This framework was developed using a lightweight consensus technique and runs on Hyperledger Fabric for both sidechain and mian chain. In vaccine production, Peng et al. (168) created an approach to blockchain technology that employs both public and private blockchains. The public blockchain, developed by Ethereum, is used for recording and verifying critical vaccine production data delivered, while the private blockchain is employed for secure information sharing among authorised participants, which is developed by HyperLedger.

Sidechains offer several advantages, including scalability by offloading specific transactions and computations from the main chain, interoperability between different blockchain networks, and the ability to experiment with new features and functionalities. However, challenges such as security, trust assumptions, and ensuring the decentralisation of Sidechain need to be carefully addressed.

#### 5.2.6 Off-chain storage

In the context of blockchain technology, storing data off the main blockchain in a separate database or decentralized storage network like IPFS, Cloud, or Swarm while still keeping the references or cryptographic proofs of that data on the blockchain is referred to as off-chain storage. In the medical industry, it offers a potentially game-changing solution to the issue of managing massive amounts of individual and medical data. This approach is crucial for overcoming the scalability issues and high storage costs associated with blockchain data storage. Although there are considerable cost and scalability benefits, customers must now trust other parties with their data. Hence, safe and verified methods of data retrieval and integrity verification are required.

As an off-chain storage mechanism, the Ethereum-based healthcare models proposed in (54, 63, 80, 83, 94) all use IPFS for image storage, while the Hyperledger Fabric-based models proposed in (50) and the personal blockchain-based models proposed in (71) both use cloud storage. In these frameworks, scalability has been achieved by using off-chain storage. While storing data off-chain can improve scalability by decreasing the burden on the blockchain network, it does come with security risks, especially for sensitive information. To address security concerns, researchers used innovative cryptographic key generation techniques. For example, trusted oracles are used for automatic key creation in (63), and a hybrid cryptosystem (which combines the public-key and symmetric-key cryptosystems) is used in (50).

In this section, we have discussed off-chain and on-chain solutions for the technical issues presented in blockchain applications. A layer-wise breakdown of these solutions has been presented in Table 8, other than optimized infrastructure. Optimized infrastructure requires changes in several layers of blockchain.

**Table 8:** Layer-wise solutions for technical issues of blockchain applications.

Blockchain layer	Tools	Proposed solutions	
Application layer	UI applications	Off-chain indexing	(155–158)
	Smart contracts	Off-chain storage	(50, 54, 71, 80, 63, 94)
		Caching	(160–162)
Middleware Layer	Libraries API’s	Off-chain Transactions	(152), (153, 154)
		Sidechain	(163–167)
Consensus layer	Consensus	Consensus optimization	(136, 137, 139, 140)
	Aalgorithms	Sharding	(143–146)
Network layer	P2P communication	Consortium blockchain	(66, 169, 170)
	Network protocols	Sharding	(143–146)
Data layer	Data infrastructure	on-chain indexing	(131, 132)

## 6 Contributions and novelty of the study

In addressing the critical need for improved healthcare data management, this review paper not only investigates the transformational potential of blockchain technology in the healthcare industry but also proposes a fresh approach to understanding its deployment. Our work makes a novel contribution by thoroughly examining solutions used in blockchain applications for healthcare. We organized and presented these ideas in a complete layer-wise implementation architecture. This framework not only explains the various layers of blockchain technology, but it also describes the specific solutions deliberately placed at each level to address issues in healthcare applications. Our layer-wise methodology, which categorizes and highlights various solutions to assist the deployment of blockchain solutions in healthcare. This unique contribution sets our research apart, providing readers with comprehensive knowledge and practical insights into the strategic use of blockchain technology to improve security, interoperability, and efficiency in healthcare data management.

## 7 Limitations and future directions

Our review paper aims to evaluate the integration of blockchain within the healthcare sector. The wide range of blockchain applications in healthcare, including data management, supply chain management, and insurance, have been discussed, but several restrictions exist. We mostly focused on performance challenges and on-chain and off-chain solutions within the blockchain framework. Furthermore, our analysis does not delve extensively into the integration of blockchain with other emerging technologies such as IoT, machine learning, artificial intelligence, and edge computing, leaving untapped potential insights into their convergence in healthcare. Future research should focus on developing interoperable frameworks that seamlessly incorporate blockchain, IoT, ML, AI, and edge computing for real-time data analysis, predictive modeling, and decentralized decision-making. Such interfaces may provide novel answers to the difficulties of scalability, interoperability, and data analytics in healthcare. Furthermore, another limitation is addressing the role of blockchain in complex environments like cross-border data transfer. In this context, questions arise about the regulatory systems and tackling the movement of healthcare data across borders globally. As outlined in Table 1, every country has its own data regulations, making the facilitation of cross-border data exchange frameworks complex. Additionally, the development of blockchain-specific regulations tailored for healthcare use presents another challenge.

Consequently, several key questions need to be addressed:

• How can ML, AI, and edge technologies work together to maximize healthcare data management while ensuring security and real-time insights?
• How might blockchain technologies help with secure and compliant cross-border data exchange, given the world’s different data protection regulations?
• What techniques may be developed to balance the decentralized benefits of blockchain with the necessity of adhering to international data transfer standards?
• What regulations are required to address the specific difficulties presented by smart contracts and decentralized data storage in healthcare?

## 8 Conclusion

In this extensive examination, blockchain technology can be used in a variety of exciting ways in the healthcare industry. The first step of the inquiry is to examine a wide range of healthcare use cases, such as drug supply chain management, clinical trial administration, EHR management, and health insurance regulation. The study uses these cases to illustrate how blockchain technology might be used to solve long-standing problems in the healthcare system. BT has the potential to drastically improve the delivery of healthcare, but it still faces the same performance challenges as other cutting-edge technologies. The problems of scalability, throughput, storage, and security stand out as particularly important ones, and they call for novel approaches to their resolution. A variety of on-chain and off-chain solutions are proposed to address these issues. Solutions are thoroughly examined both on-chain and off-chain. Subsequently, we demonstrated layer-by-layer implementations to improve understanding and ease real-time implementation in blockchain healthcare applications. Additionally, we provided feature directions for our study. The purpose of this analysis is to provide a reference point for those working on blockchain-based healthcare applications in the future.
